# Supplementary material for: Blood Endothelial-Cell Extracellular Vesicles as Potential Biomarkers for the Selection of Plasma in COVID-19 Convalescent Plasma Therapy
Source: Cells. 2022 Oct 4;11(19):3122. doi: 10.3390/cells11193122 (PMC9563445; doi:10.3390/cells11193122)
Supplement: Supplementary file 1 [file cells-11-03122-s001.zip › cells-1904713-supplementary.pdf]

Blood endothelial cell extracellular vesicles as potential biomarkers for the selection of plasma in COVID-19 convalescent plasma therapy

Supplementary material

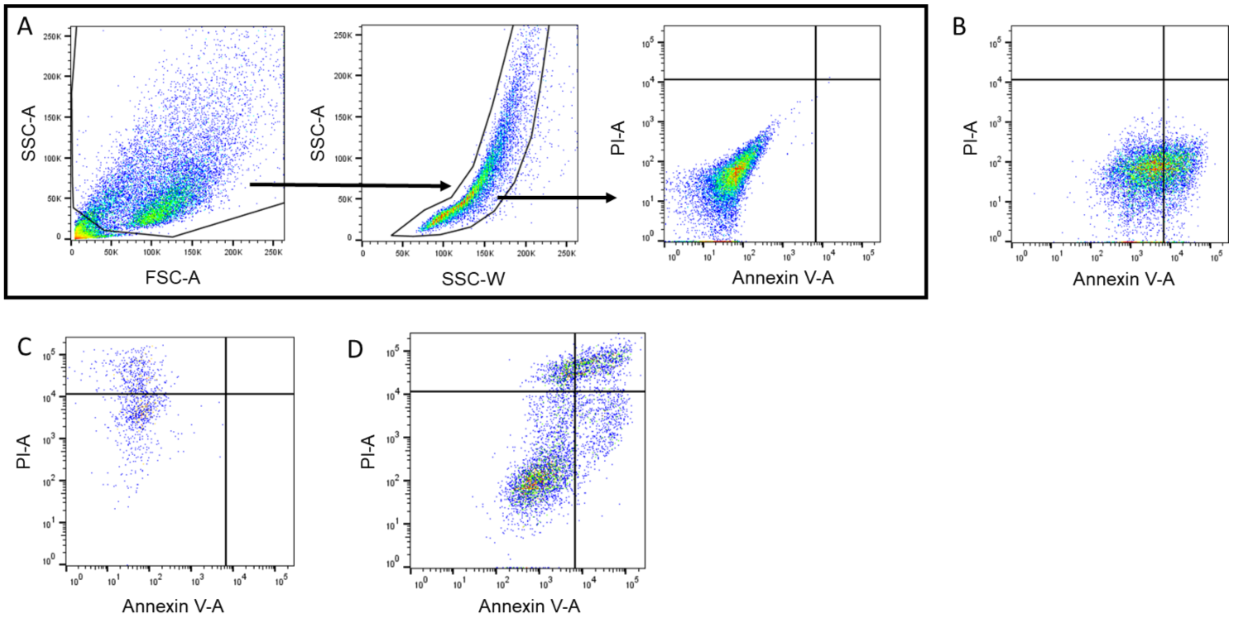

Figure S1. Flow cytometry gating strategy for the measurement of cell viability. (A) Cells were first selected based on their size and granularity. Single cells were then selected, and gates were placed using the unstained control. Based on single stained cells for (B) Annexin V450 and (C) propidium iodide, the appropriate gates were established to descriminate between early and late apoptosis, and necrosis. (D) Cells treated with staurosporin (2µm) were used as a positive control for cell death.

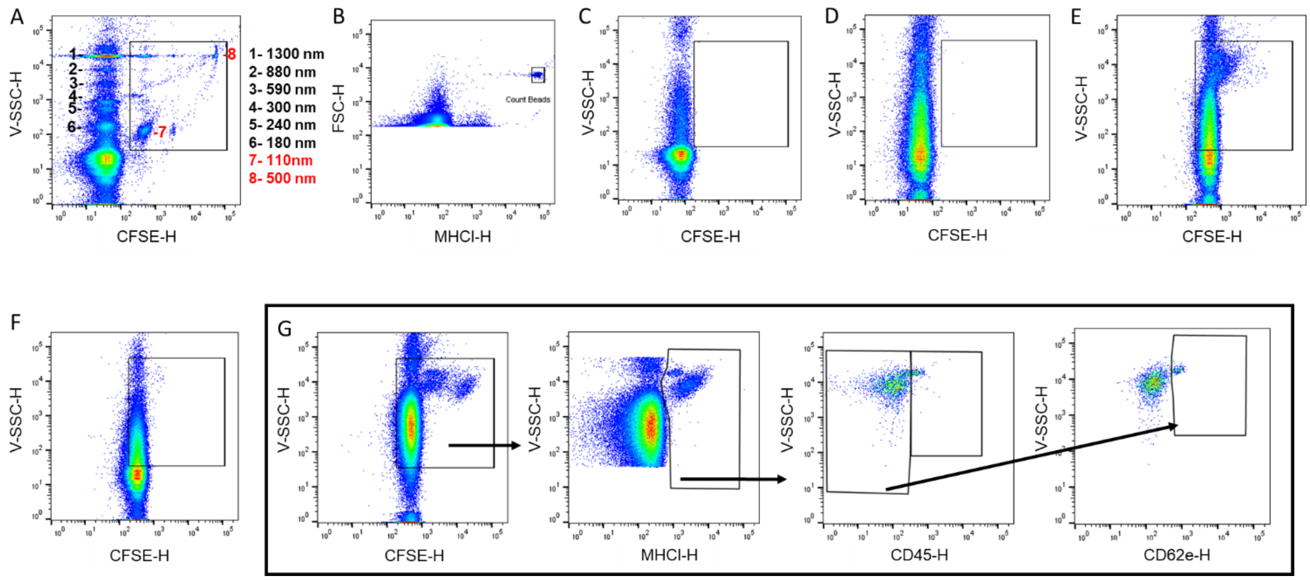

Figure S2. Flow cytometry gating strategy for EVs. (A) The FACSCelesta was first calibrated for EV detection using the ApogeeMix (Apogee Flow Systems), a mixture of non-fluorescent silica beads (180, 240, 300, 590, 880, and 1300 nm, in black) and FITC-fluorescent latex beads (110 and 500 nm, in red), and (B) count beads (diameter= 3um). (C) A sample without any vesicles, but with the antibody mix and the count beads, were used to subtract background (noise) from every samples. (D) An unstained sample containing the vesicles, but no antibody and (E) a single-stained sample were also used for compensations between fluorochromes. (F) Triton X-100 was also added in the sample tubes at the end of the experiments to confirm the presence of vesicles from cellular origin. (G) Gating strategies for BEC-EVs analysis are depicted in the black rectangle. FACS plots and histograms are showing all parameters in height (indicated as –H), as recommended for EV detection. SSC, side scatter; FSC-H, forward scatter-height; CFSE, carboxyfluorescein succinimidyl ester; MHCI, major histocompatibility complex I.

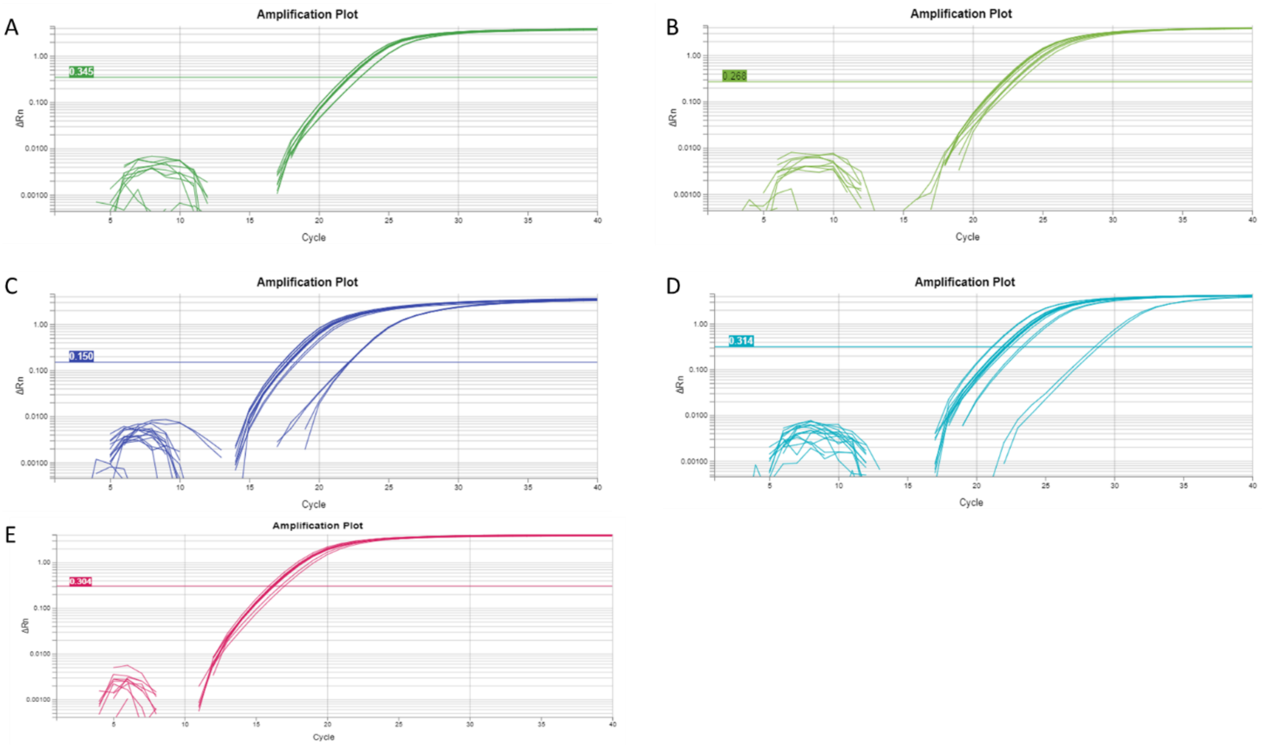

Figure S3. Amplification plot for (A) hNDUFA9 (B) hUQCRC2 (C) hICAM1 (D) hNFkB (E) hACTB qPCR analysis.

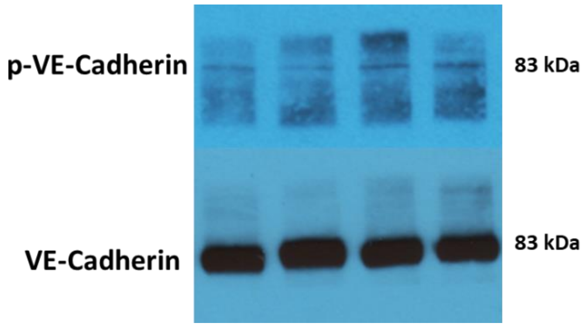

Figure S4. Immunoblotting for phospho VE-Cadherin and VE-Cadherin.
